# Multi-omics revealed the long-term effect of ruminal keystone bacteria and the microbial metabolome on lactation performance in adult dairy goats

**DOI:** 10.1186/s40168-023-01652-5

**Published:** 2023-09-29

**Authors:** Dangdang Wang, Luyu Chen, Guangfu Tang, Junjian Yu, Jie Chen, Zongjun Li, Yangchun Cao, Xinjian Lei, Lu Deng, Shengru Wu, Le Luo Guan, Junhu Yao

**Affiliations:** 1 College of Animal Science and Technology, Northwest A&F University, Yangling, Shaanxi 712100 People’s Republic of China; 2 Key Laboratory of Livestock Biology, Northwest A&F University, Yangling, Shaanxi 712100 People’s Republic of China; 3 Department of Agricultural, Food and Nutritional Science, University of Alberta, 116 St. and 85 Ave, Edmonton, AB Canada

**Keywords:** Rumen microbiome, Microbial interaction, Keystone bacteria, Rumen metabolome, Long-term effects and prediction, Goats

## Abstract

**Background:**

The increased growth rate of young animals can lead to higher lactation performance in adult goats; however, the effects of the ruminal microbiome on the growth of young goats, and the contribution of the early-life rumen microbiome to lifelong growth and lactation performance in goats has not yet been well defined. Hence, this study assessed the rumen microbiome in young goats with different average daily gains (ADG) and evaluated its contribution to growth and lactation performance during the first lactation period.

**Results:**

Based on monitoring of a cohort of 99 goats from youth to first lactation, the 15 highest ADG (HADG) goats and 15 lowest ADG (LADG) goats were subjected to rumen fluid microbiome and metabolome profiling. The comparison of the rumen metagenome of HADG and LADG goats revealed that ruminal carbohydrate metabolism and amino acid metabolism function were enhanced in HADG goats, suggesting that the rumen fluid microbiome of HADG goats has higher feed fermentation ability. Co-occurrence network and correlation analysis revealed that *Streptococcus*, *Candidatus Saccharimonans*, and *Succinivibrionaceae UCG-001* were significantly positively correlated with young goats’ growth rates and some HADG-enriched carbohydrate and protein metabolites, such as propionate, butyrate, maltoriose, and amino acids, while several genera and species of *Prevotella* and Methanogens exhibited a negative relationship with young goats’ growth rates and correlated with LADG-enriched metabolites, such as rumen acetate as well as methane. Additionally, some functional keystone bacterial taxa, such as *Prevotella*, in the rumen of young goats were significantly correlated with the same taxa in the rumen of adult lactation goats. *Prevotella* also enriched the rumen of LADG lactating goats and had a negative effect on rumen fermentation efficiency in lactating goats. Additional analysis using random forest machine learning showed that rumen fluid microbiota and their metabolites of young goats, such as *Prevotellaceae UCG-003*, acetate to propionate ratio could be potential microbial markers that can potentially classify high or low ADG goats with an accuracy of prediction of > 81.3%. Similarly, the abundance of *Streptococcus* in the rumen of young goats could be predictive of milk yield in adult goats with high accuracy (area under the curve 91.7%).

**Conclusions:**

This study identified the keystone bacterial taxa that influence carbohydrate and amino acid metabolic functions and shape the rumen fluid microbiota in the rumen of adult animals. Keystone bacteria and their effects on rumen fluid microbiota and metabolome composition during early life can lead to higher lactation performance in adult ruminants. These findings suggest that the rumen microbiome together with their metabolites in young ruminants have long-term effect on feed efficiency and animal performance. The fundamental knowledge may allow us to develop advanced methods to manipulate the rumen microbiome and improve production efficiency of ruminants.

Video Abstract

**Supplementary Information:**

The online version contains supplementary material available at 10.1186/s40168-023-01652-5.

## Background

There is an increasing demand to produce goat milk because it consists of more medium- and short-chain fatty acids, vitamins, β-casein, and trace minerals, and less α-casein and allergens than dairy cows’ milk and is more suitable for infant nutrition [1, 2]. Additionally, goat milk and its products provide important daily food sources of protein, phosphate, and calcium for people in the developing world [3]. Many factors can affect dairy goat milk production and quality, including genetics [4, 5], management [6], and feeding strategy [7]. The rumen serves as a bioreactor that enables animals to digest complex plant fibers and polysaccharides and produce volatile fatty acids (VFAs), microbial proteins, and vitamins [8]. Recent research has revealed that the rumen microbiome plays a vital role in affecting production traits of dairy cows, such as feed efficiency [9, 10], methane yield [11, 12], and milk production [13, 14]. However, the contribution of the rumen microbiome to lactation performance in goats is less understood.

Early-life rumen microbiome plays key roles in rumen development and microbial fermentation, which subsequently affects the growth and feed efficiency of young ruminants [15–17]. The VFAs produced by the rumen microbiome are vital to stimulate rumen tissue metabolism and epithelium development during early life [18]. It has been reported that rumen microbiota transplantation could alter the endogenous microbiota and lead to improved growth performance in calves and lambs [19–21]. Importantly, the preweaning and prepubertal ADG of heifers or goat kids have been reported to be highly correlated with adult milk production [22–24]. Similarly, a study revealed that the initial microbiota after birth has a long-lasting effect on the assembly process and adult composition of the ruminal microbiome based on the observations during 3 years of the cows’ life [25]. Early-life dietary interventions in dairy calves can also affect microbial colonization, with long-term consequences for the adult cow rumen microbiota and fermentation [26]. Although recent studies have remarkably expanded our understanding of the early-life rumen microbiome, there is a knowledge gap about whether and how highly efficient phenotypes (e.g., growth performance)-related ruminal microbiota in the early or adolescent life of goats could affect microbial community dynamic succession, and subsequently affect host lactation performance. In addition, a comprehensive understanding of the mechanisms of the rumen microbiome driving growth in dairy goats is still lacking.

The ruminal microbiota composition in young ruminants on subsequent ruminal microbiota succession in adult ruminants may serve as the potential mechanism to determine the link between the higher growth performance, such as ADG, in the youth period and the higher lactation performance in the adult period. In the present study, 6-month-old dairy goats with different ADG were used as a model to monitor their growth and performance until first lactation and to assess the rumen fluid microbiota, microbiome, and metabolome using metataxonomics, metagenomics, and metabolomics, respectively, aiming to (i) identify the contribution of the rumen fluid microbiome and their metabolome to the growth performance of young goats; (ii) identify whether the rumen fluid microbiota of young goats has a long-term effect on rumen microbiota colonization of adult goats and then contributes to regulating lactation performance; and (iii) explore rumen fluid microbes during the youth period that can be used as biological markers for predicting future milk production.

## Results

### Young dairy goats had differential growth rates and rumen fermentation

**Fig. 1:**
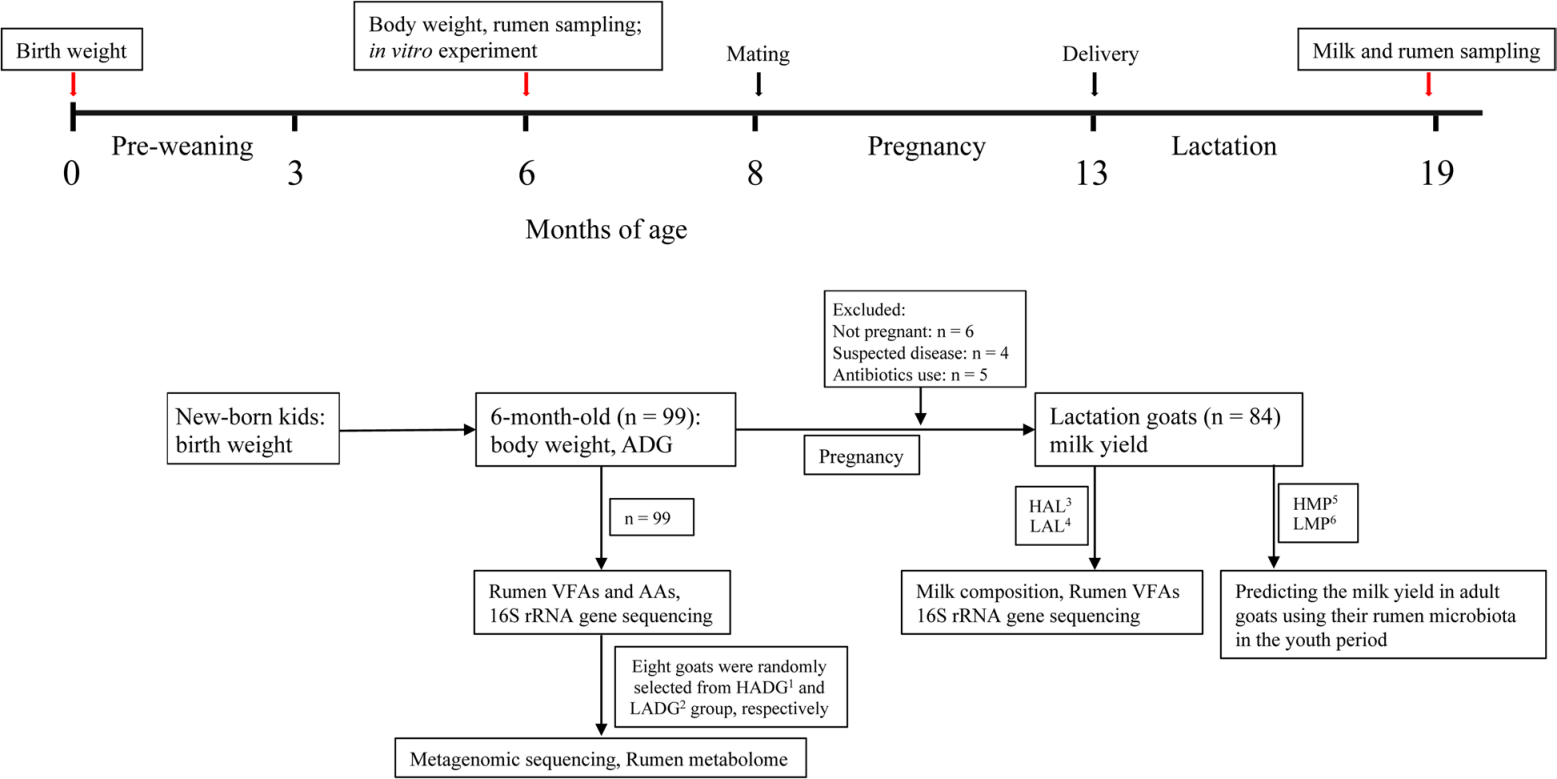
The flowchart of this study. All animals were fed the same diet, kept under the same conditions, and housed together from birth (Table S9). During the preweaning phase, all kids were fed milk, alfalfa hay and concentrate mixture, and the weaned animals (3 months old to ~ 13 months old) were fed total mixed ration (TMR) with a forage to concentrate ratio of 60:40. After delivery, goats were fed TMR with forage:concentrate rations of 50:50. ^1,2^ From 99 young goats enrolled in this study, 15 goats with the highest average daily gain (ADG) were selected as the HADG group (ADG: 132.5 ± 1.5 g/day); 15 goats with the lowest ADG were selected as the LADG group (ADG: 88.2 ± 1.2 g/day). ^3,4^ After delivery of their newborn offspring kids, 84 of 99 healthy lactating goats remained, and 15 goats were excluded. In the HAL group, 13 lactating goats of 15 HADG goats remained and were renamed the HAL group; in the LAL group, 12 lactating goats of 15 LADG goats remained and were renamed the LAL group. ^5,6^ Among these 84 lactating goats, 15 lactating goats with the highest milk yield were selected as the HMP group (average daily milk yield: 2.82 ± 0.04 kg/day), and 15 lactating goats with the lowest milk yield were selected as the LMP group (average daily milk yield: 1.51 ± 0.03 kg/day), according to the milk yield recorded every 7 days over the first whole lactation period ADG: average daily gain, AA: amino acid, HADG: young goats with high average daily gain, LADG: young goats with low average daily gain, HAL: lactating goats of HADG, LAL: lactating goats of LADG, HMP: lactating goats with high milk yield, LMP: lactating goats with low-milk yield

This study monitored the growth performance of 99 young goats, 84 of which successfully entered the lactation phase (Fig. 1). The growth performance parameters of 99 young dairy goats (187.9 ± 0.3 days of age [mean ± standard error (SE)], Table S1), including body weight (23.6 ± 0.3 kg, [coefficient of variation (CV)] = 12.01%), weight gain (20.6 ± 0.3 kg, [CV] = 13.00%), and ADG (109.47 ± 1.46 g/day, CV = 13.21%), were highly correlated (Spearman’s correlation coefficient > 0.97, *p* < 0.01).

**Fig. 2:**
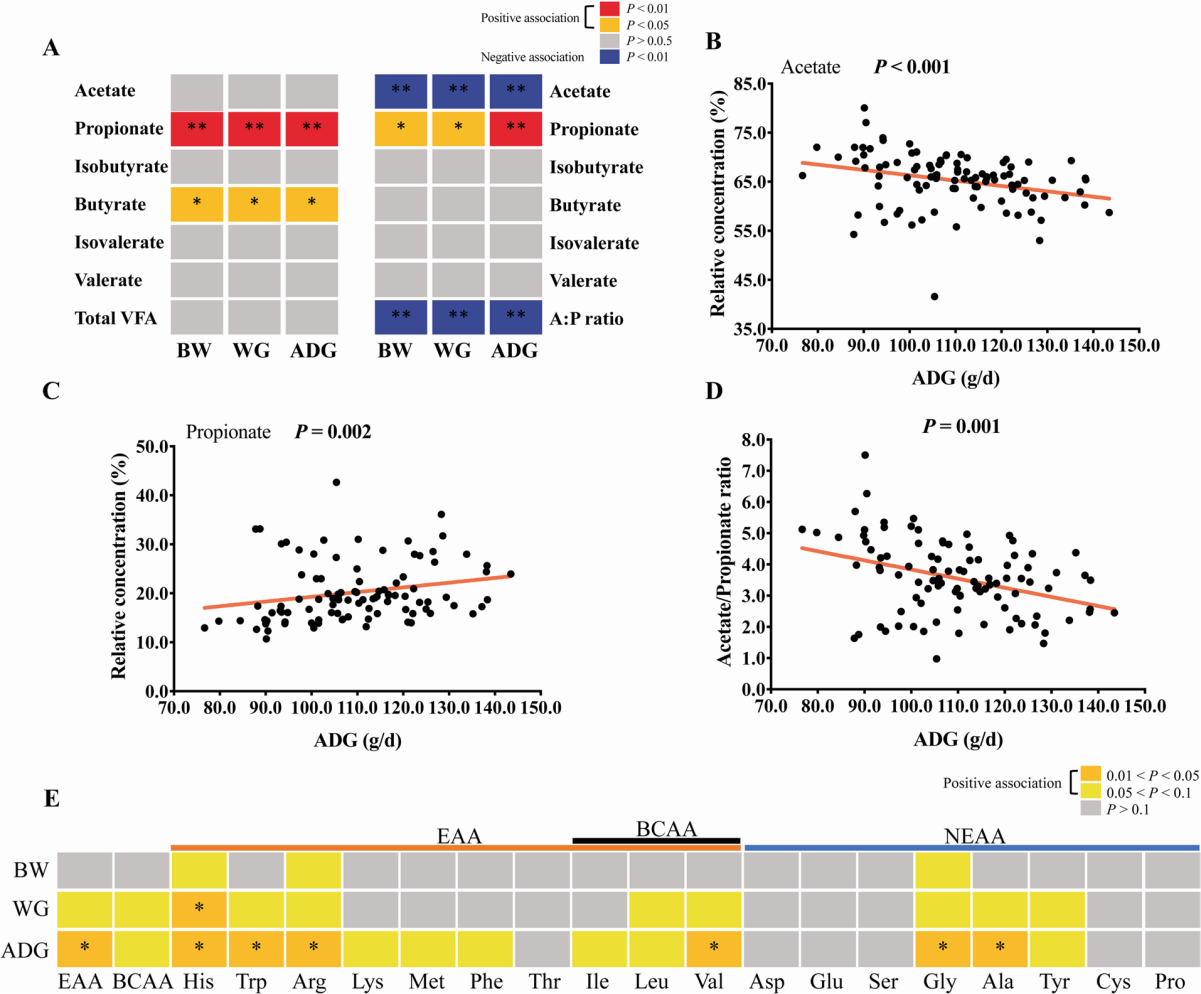
Rumen fermentation productions and their relationship with growth performance traits (*n* = 99). **A** Associations between rumen VFAs and growth performance traits. Left: absolute concentrations of VFAs, Right: relative abundance of VFAs. The molar percentage of acetate (**B**), propionate (**C**), and the acetate to propionate ratio (**D**) were significantly associated with ADG (Spearman’s correlation, *p* < 0.05). **E** Heatmap showing the association between rumen fluid free amino acids and growth performance traits (Spearman’s correlation, *p* < 0.05). *p < 0.05, **p < 0.01 BW: body weight, WG: weight gain, ADG: average daily gain, EAA: essential amino acid, BCAA: branched chain amino acid, NEAA: non-essential amino acid

The analysis of the relationship between growth performance traits and rumen VFA profiles (*n* = 99) showed that several VFAs were significantly associated with these growth performance traits (Fig. 2a–d; Table S2). Specifically, rumen propionate and butyrate concentrations were positively correlated with body weight, weight gain, and ADG (*r* > 0.20, *p* < 0.05; Fig. 2a). The molar percentage of propionate was positively correlated with body weight, weight gain, and ADG (*r* > 0.25, *p* < 0.05; Fig. 2a, c), while the molar percentage of acetate, acetate to propionate ratio were negatively correlated with these traits (*r* <  − 0.28, *p* < 0.01; Fig. 2a, b, d). Analysis of Spearman correlations between growth performance traits and rumen free amino acids measurements (*n* = 99) showed that ADG was significantly positively correlated with the rumen fluid concentrations of total essential amino acids, His, Trp, Arg, Val, Gly, and Ala (*r* > 0.2, *p* < 0.05; Fig. 2e). ADG had a weak positive correlation with the rumen fluid concentrations of total branched chain amino acids, Lys, Met, Phe, Ile, Leu, and Tyr (*p* < 0.1).

### Varied ruminal microbiota diversity and key bacteria related to growth rates in young dairy goats

**Fig. 3:**
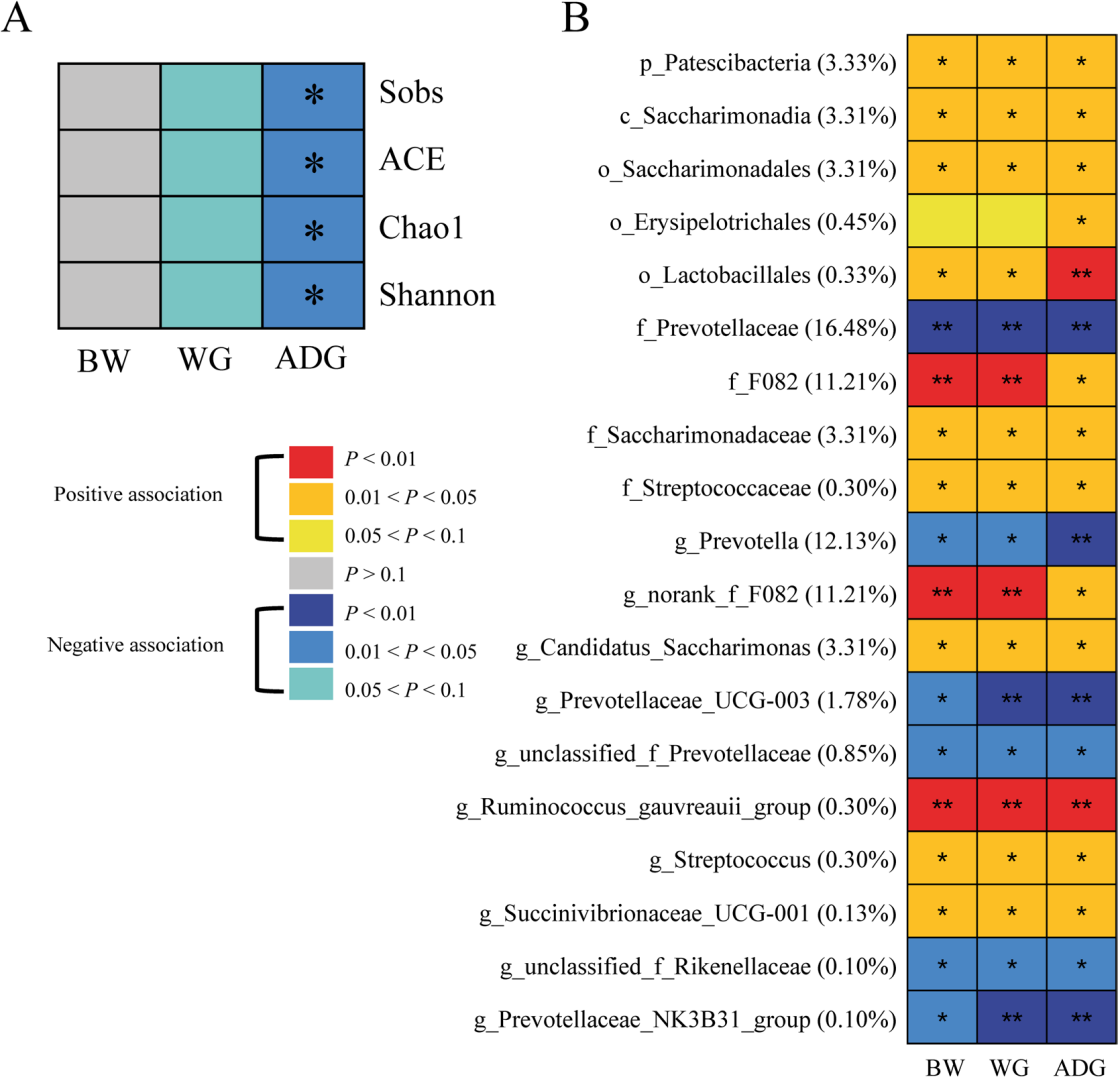
The relationship between rumen fluid microbiota features and goat growth performance traits (*n* = 99). **A** Associations between alpha diversity of ruminal microbiota and growth performance traits. **B** Heatmap showing the association between bacterial taxa (average relative abundance ≥ 0.1%) and growth performance traits (Spearman’s correlation, *p* < 0.05, *r* >|0.2|), % the relative abundance of the taxa. *p < 0.05, **p < 0.01 BW: body weight, WG: weight gain, ADG: average daily gain

The metataxonomic analysis of rumen fluid samples collected from young dairy goats (*n* = 99) using 16S rRNA gene amplicon sequencing revealed a moderate negative association (*r* <  − 0.20, *p* < 0.05) between ADG and Sobs, ACE, Chao1, and Shannon indices (Fig. 3a). The correlation between rumen fluid bacteria and growth performance traits (body weight, weight gain, and ADG) of young dairy goats (*n* = 99) was also identified (Fig. 3b; Table S3). At the family level (relative abundance ≥ 0.1%), the abundances of *F082*, *Saccharimonadaceae*, and *Streptococcaceae* were positively correlated with body weight, weight gain, and ADG, while *Prevotellaceae* were negatively correlated with these growth performance traits of young goats (*r* >|0.2|, *p* < 0.05). Among the bacterial genera (relative abundance ≥ 0.1%), the abundances of *norank F082*, *Candidatus Saccharimonas*, *Ruminococcus gauvreauii* group, *Streptococcus*, and *Succinivibrionaceae UCG-001* showed significant positive associations with body weight, weight gain, and ADG, and the abundances of *Prevotella*, *Prevotellaceae UCG-003*, unclassified *Prevotellaceae*, unclassified *Rikenellaceae*, and *Prevotellaceae NK3B31* group showed significant negative associations with these growth performance traits (*r* >|0.2|, *p* < 0.05).

From this animal cohort (*n* = 99), two groups of goats with divergent ADG were identified: high ADG (HADG, *n* = 15) and low ADG (LADG, *n* = 15) (132.5 ± 1.5 g/day vs 88.2 ± 1.2 g/day, *p* < 0.001). When the rumen fluid microbiota was further compared between HADG and LADG goats, the rumen microbial community in HADG goats had lower alpha diversity indices (*p* < 0.05; Fig. S1a) and was clearly distinguished from that in LADG goats (ANOSIM *R* = 0.244, *p* = 0.001; Fig. S1b). In addition, compared to the LADG goats, the relative abundances of family *F082*, *Saccharimonadaceae*, *Streptococcaceae*, genus *norank F082*, *Candidatus Saccharimonas*, *Ruminococcus gauvreauii* group, *Streptococcus*, and *Succinivibrionaceae UCG-001* were significantly higher in the rumen of HADG goats (abundance relative ≥ 0.1%; *p* < 0.05; Fig. S1c, d). In contrast, family *Prevotellaceae*, genus *Prevotella*, *Prevotellaceae UCG-003*, unclassified *Prevotellaceae*, and unclassified *Rikenellaceae* were significantly higher in LADG goats (abundance relative ≥ 0.1%; *p* < 0.05; Fig. S1c, d).

### Microbial interactions differed between high and low performance goats

**Fig. 4:**
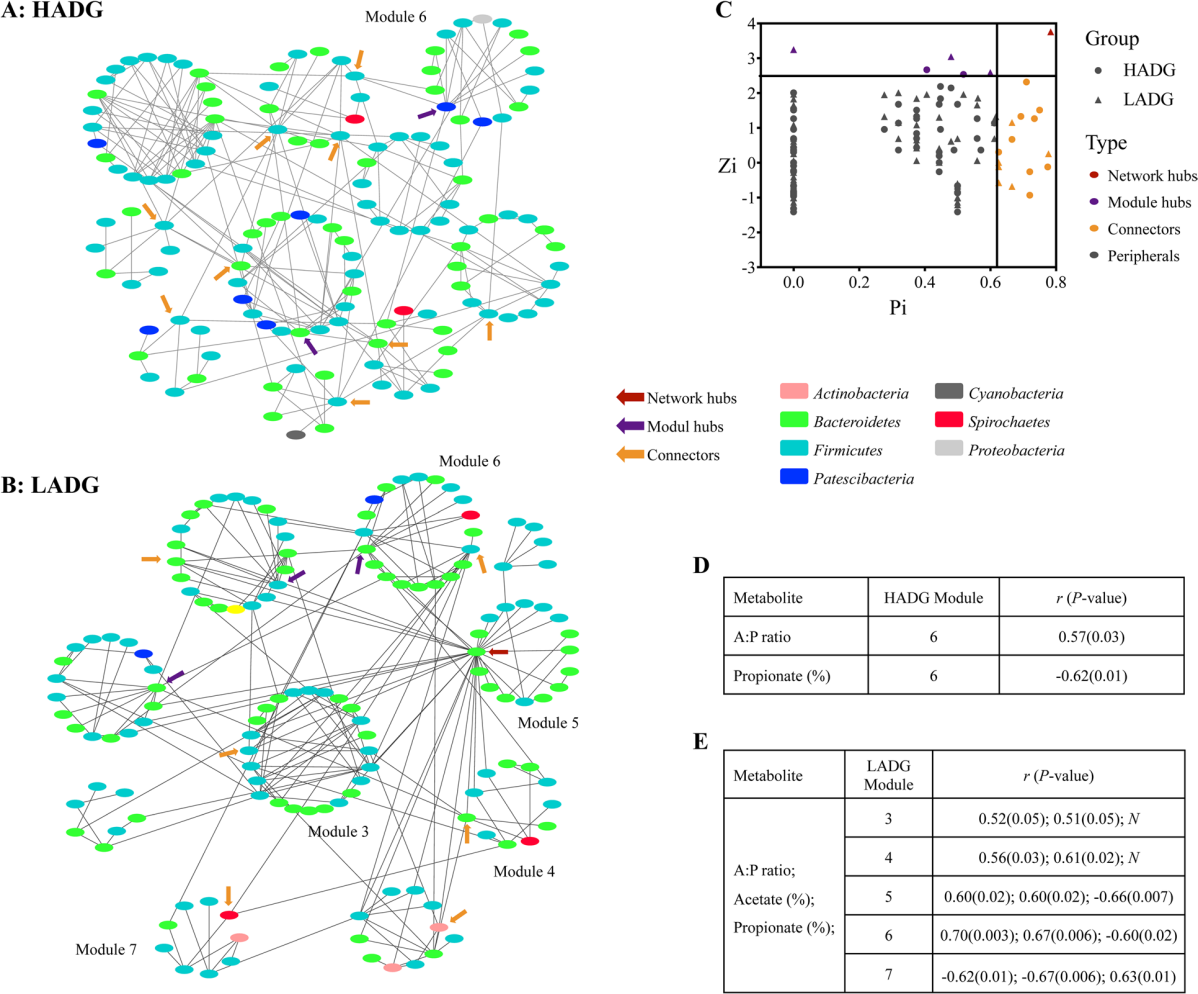
Co-occurrence network of ASVs in HADG and LADG goats, and network modules associated with rumen VFAs. Co-occurrence network of ASVs in HADG (**A**) and LADG goats (**B**). Nodes represent an ASV, and only significant (Pearson’s correlation, *p* < 0.05) relationships are shown in solid lines.** C** The scatter plot shows the distribution of ASVs based on their network roles. Network modules associated with rumen VFAs in HADG (**D**) and LADG goats (**E**). *N*: no significant difference HADG: young goats with high average daily gain, LADG: young goats with low average daily gain, Zi: within-module connectivity, Pi: among-module connectivity

As bacteria-bacteria interactions are key modulators that shape the rumen microbiota, we further evaluated the potential microbial interactions (Fig. 4a, b) and identified the keystone bacterial taxa (Fig. 4c; Table S4) in the rumen fluid microbiota of HADG and LADG goats using random matrix theory (RMT)-based network analysis. A total of two nodes were highly connected species within their own module (a high Zi > 2.5 and a low Pi ≤ 0.62), which may act as module hubs in the HADG network (Fig. 4c; Table S4). ASV752 assigned to *Candidatus Saccharimonas*, and ASV4676 assigned to *Oscillospiraceae NK4A214* group, may act as module hubs. Nine nodes had low Zi (≤ 0.25) and high Pi (> 0.62) values, linked several modules together and acted as connector species in the HADG network (Fig. 4c; Table S4). For example, ASV806, and ASV1105 were annotated to *Oscillospiraceae NK4A214* group, and ASV4527 was annotated to *Christensenellaceae R-7* group, which may act as connectors in the HADG network. In addition, three module hubs, two of which were annotated to *Prevotellaceae UCG-003* and *Christensenellaceae R-7* group, were identified in the LADG network. Notably, ASV2928 (annotated to *Prevotella*) had high Zi (> 2.5) and Pi (> 0.62) values, was highly connected species within its own module and linked several modules together, acting as a supergeneralist (network hub) that was detected in the LADG network (Fig. 4c; Table S4).

Correlation analyses between module-based eigengenes and rumen VFAs were conducted to understand the relationship between individual modules and rumen fermentation (Fig. 4d, e). In the rumen microbial community of the LADG goats, at least five modules were significantly correlated with the VFAs or one of their two major components, acetate, and propionate. Module 3, 4, 5, and 6 were positively correlated with both the molar percentage of acetate, and the acetate to propionate ratio (*r* > 0.52, *p* < 0.05), while the latter two modules were negatively correlated with the molar percentage of propionate (*r* < -0.60, *p* < 0.05). Notably, the genus *Prevotella* was a predominant feature in module 3, including the network hub (ASV2928). In addition, 4 out of 11 or 4 out of 16 nodes in modules 4 and 5 were assigned to the family *Prevotellaceae*. In the HADG network, only module 6 was negatively associated with the molar percentage of propionate, and positively correlated with the acetate to propionate ratio (*r* >|0.57|, *p* < 0.05).

### Functional variation in the rumen fluid microbiome related to the growth performance of young goats

We then compared the functional capacities of the rumen fluid microbiome between goats with high (*n* = 8) and low (*n* = 8) growth performance using metagenomic analysis. From a total of 1,200,051,228 metagenome reads (75,003,202 ± 965,517 per sample), 10,278,318 contigs were annotated (642,395 ± 26,180 per sample).

**Fig. 5:**
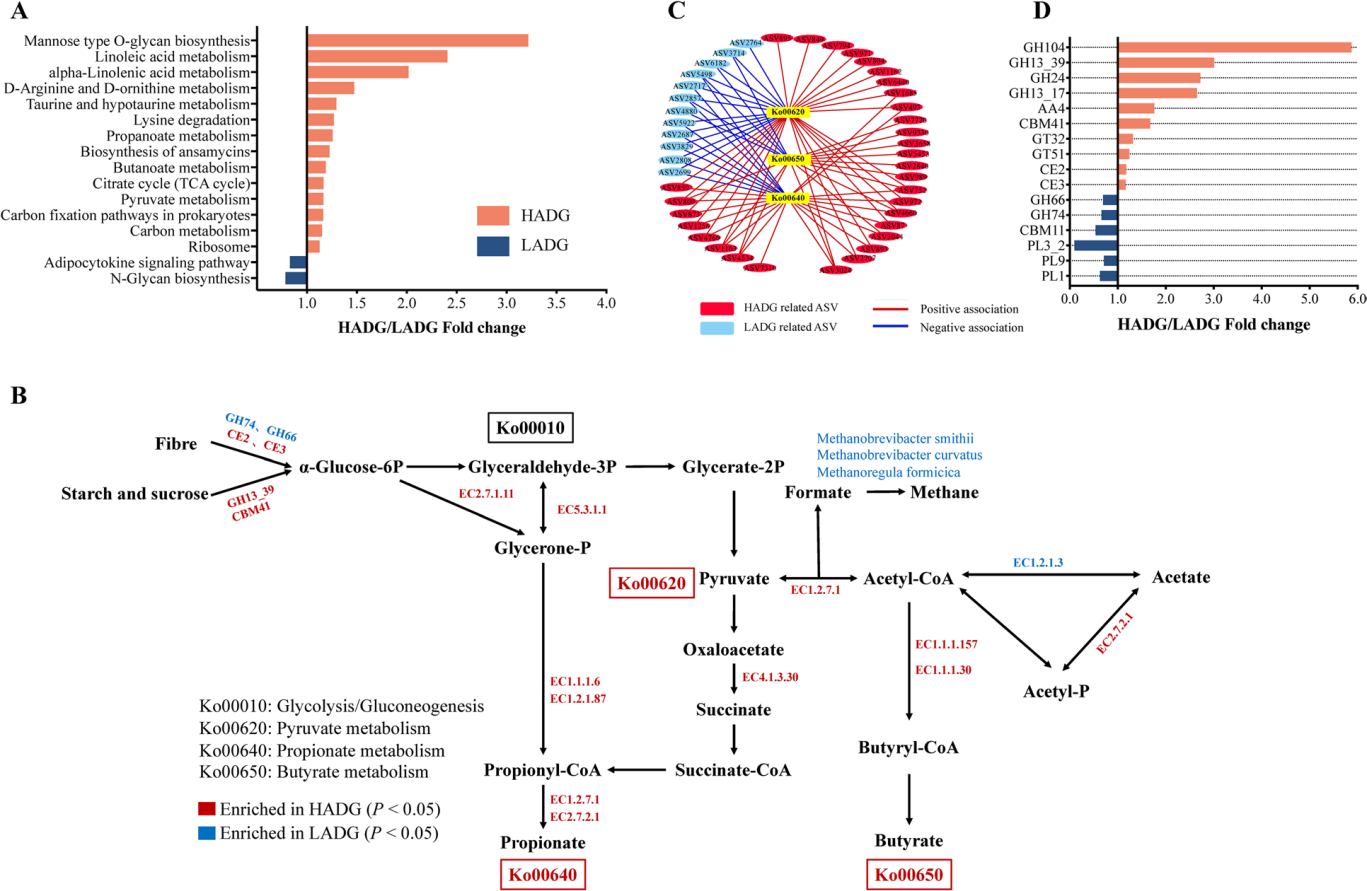
Difference in functional capacities of the rumen microbiome between HADG and LADG goats (*n* = 8 each group). **A** Differences in level 3 KEGG microbial pathways between HADG and LADG goats (Wilcoxon rank-sum test, *p* < 0.05).** B** Reconstruction of the metabolic pathway associated with VFA biosynthesis and methanogenesis.** C** Spearman’s rank correlations between the ADG-associated ASVs and the VFA-related KEGG pathways (*p* < 0.05).** D** Differential CAZyme functions between HADG and LADG goats based on family-level enzymes (Wilcoxon rank-sum test, *p* < 0.05) HADG: young goats with high average daily gain, LADG: young goats with low average daily gain

In total, 16 significantly different KEGG pathways were identified, 14 of which were enriched in the rumen microbiome of HADG goats (*p* < 0.05; Fig. 5a). Among them, 13 pathways were related to “metabolism” (first-level KEGG functions), including four “carbohydrate metabolism” pathways (TCA cycle, pyruvate metabolism, butanoate metabolism, and propionate metabolism), three “amino acid metabolism” pathways (taurine and hypotaurine metabolism, lysine degradation, and D-arginine and D-ornithine metabolism), two “lipid metabolism” pathways (linoleic acid metabolism, and alpha-linoleic acid metabolism), “energy metabolism” pathway (carbon fixation pathways in prokaryotes), “global and overview map” pathway (carbon metabolism), and “glycan biosynthesis and metabolism” pathway (mannose type O-glycan biosynthesis). In the rumen fluid microbiome of LADG goats, N-glycan biosynthesis and adipocytokine signaling pathways were enriched.

Because 4 out of 16 differentially abundant KEGG pathways were “carbohydrate metabolism,” which produce rumen VFAs, the main energy source, we then analyzed genes encoding enzymes involved in the metabolic pathways associated with VFA biosynthesis (glycolysis/gluconeogenesis, pyruvate metabolism, propionate metabolism, and butyrate metabolism). Of these identified enzymes that were significantly enriched in the rumen of HADG goats (*p* < 0.05; Fig. 5b; Table S5), 6-phosphofructokinase (EC2.7.1.11) and triose-phosphate isomerase (EC5.3.1.1) were involved in translating glucose-6P to pyruvate. Glycerol dehydrogenase (EC1.1.1.6) and propanal dehydrogenase (CoA-propanoylating, EC1.2.1.87) were involved in translating glycerone-P to propanoyl-CoA. Furthermore, methylisocitrate lyase (EC4.1.3.30), pyruvate synthase (EC1.2.7.1), and acetate kinase (EC2.7.2.1) were involved in translating pyruvate to propionate. Notably, methylisocitrate lyase (EC4.1.3.30), which converts methylisocitrate to succinate, was significantly higher in the HADG group. The results showed that 3-hydroxybutyryl-CoA dehydrogenase (EC1.1.1.157), and 3-hydroxybutyrate dehydrogenase (EC1.1.1.30), which can translate acetyl-CoA to butyrate, were also significantly higher in the HADG group. In contrast, aldehyde dehydrogenase (NAD +) (EC1.2.1.3), which is involved in translating acetyl-CoA to acetate, was enriched in the rumen microbiome of LADG goats (*p* = 0.031; Fig. 5b; Table S4). A Spearman’s rank correlation network further revealed varied relationships between ADG-associated ASVs and KEGG pathways (pyruvate metabolism, propionate metabolism, and butanoate metabolism) in the rumen fluid microbiome of HADG and LADG goats. A total of 43 ADG-related ASVs showed a significant correlation with these three pathways; 31 HADG-enriched ASVs, including some belonged to *Candidatus Saccharimonas* and *Ruminococcus gauvreauii*, had a positive correlation with these three pathways; and 12 LADG-enriched ASVs, such as ASV2717 belonged to *Prevotellaceae* UCG-003, had negative relationships with the propionate pathway and butyrate pathway (Fig. 5c; Table S6).

In addition, a total of 16 significantly differential CAZyme gene families were identified between the rumen fluid microbiome of HADG and LADG goats (*p* < 0.05; Fig. 5d). Among them, 10 CAZymes that were involved in the degradation of starch (such as GH13_39 and CBM41), xylan (CE2 and CE3), lignin (AA4), and peptidoglycan (GH24 and GH104) were enriched in the rumen fluid microbiome of HADG goats, while 6 CAZymes that were involved in the metabolism of cellulose (GH74 and GH66) and pectin (PL1, PL3_2 and PL9) were enriched in the rumen fluid microbiome of LADG goats.

### Methanogenesis differed between high and low ADG goats

The metagenomics analysis revealed that several methane production-related archaeal species, including *Methanobrevibacter smithii*, *Methanobrevibacter curvatus*, and *Methanoregula formicica*, were significantly higher in the rumen of LADG goats (*p* < 0.05; Fig. 5b; Fig. S2). We further verified the effects of the rumen fluid microbiome on rumen fermentation and methane production using in vitro incubation analysis using rumen fluid from HADG and LADG young goats (*n* = 15 each group). The results showed that HADG goats produced significantly more propionate, less molar percentage of acetate, less acetate to propionate ratio, and less methane than LADG animals (*p* < 0.05; Fig. S3).

### Rumen metabolome profiling differed between high and low performance goats

**Fig. 6:**
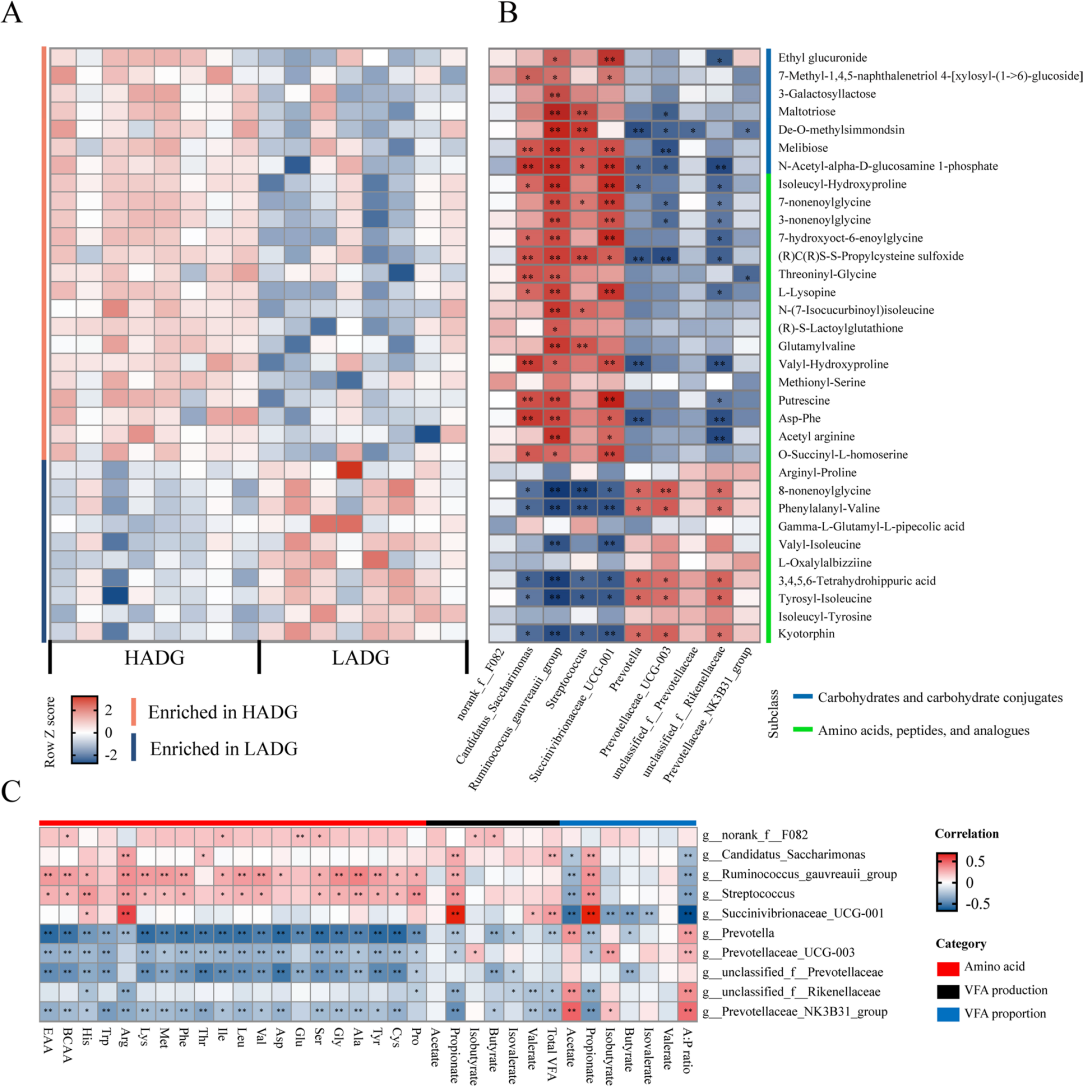
The relationship between the ADG-associated microbiota and ruminal metabolites.** A** Ruminal metabolites that differed in normalized abundance between HADG and LADG goats (*n* = 8 each group) and were significantly associated with ADG (Spearman’s correlation, *p* < 0.05). **B** Correlations between the ADG-associated metabolites and the ADG-associated microbiota (*n* = 99, Spearman’s correlation). * *p* < 0.05, ** *p* < 0.01 HADG: young goats with high average daily gain, LADG: young goats with low average daily gain

Further comparison of rumen metabolome profiles between HADG and LADG goats revealed 43 metabolites belonged to carbohydrates and carbohydrate conjugates, and amino acids, peptides, and analogs showed significant differences between HADG and LADG (*p* < 0.05; Table S7). Of these, 27 metabolites were significantly higher in the rumen of HADG goats, and the relative concentrations of 16 metabolites were significantly higher in the rumen of LADG goats (*p* < 0.05; Table S7). Then, correlation analysis between these different metabolome data and ADG was performed using Spearman’s rank correlation. The results showed that 33 metabolites were significantly associated with ADG (*r* >|0.5|, *p* < 0.05; Fig. 6a); 23 of them were significantly higher in HADG and positively associated with ADG, including L-lysopine, glutamylvaline, isoleucyl-hydroxyproline, threoninyl-glycine, valyl-hydroxyproline, methionyl-serine, and O-succinyl-l-homoserine, which belonged to amino acids, short peptides and analogs, and maltoriose, melibiose, and 3-galactosyllactose, which belonged to carbohydrates and carbohydrate conjugates (HADG-associated metabolites, *p* < 0.05). Meanwhile, 10 metabolites, including pheylalanyl-valine, valyl-isoleucine, and tyrosyl-isoleucine, were significantly higher in LADG and negatively associated with ADG (LADG-associated metabolites, *p* < 0.05).

The correlation analysis between ADG-related taxa and differential ruminal fermentation measures (Fig. 6c) showed that HADG-associated bacteria (*Candidatus Saccharimonas*, *Ruminococcus gauvreauii* group, *Streptococcus*, and *Succinivibrionaceae UCG-001*) were positively correlated with the concentration of propionate, and the molar percentage of propionate, and were negatively correlated with the molar percentage of acetate, and the acetate to propionate ratio (*r* >|0.2|, *p* < 0.05). Notably, the abundance of *Ruminococcus gauvreauii* group, *Streptococcus* also had a positive association with rumen amino acid concentration (*r* >|0.2|, *p* < 0.05). LADG-associated bacteria (*Prevotella*, *Prevotellaceae UCG-003*, *unclassified Rikenellaceae*, *Prevotellaceae NK3B31* group) were positively correlated with the molar percentage of acetate, the acetate to propionate ratio, were negatively correlated with the concentration of propionate, and the molar percentage of propionate (*r* >|0.2|, *p* < 0.05); *Prevotella*, *Prevotellaceae UCG-003*, unclassified *Prevotellaceae*, and *Prevotellaceae NK3B31* group were negatively correlated with the concentration of amino acids (*r* >|0.2|, *p* < 0.05).

Further analysis between ADG-associated microbiota and ADG-associated rumen metabolome (Fig. 6b) revealed that some HADG-enriched bacteria, such as *Ruminococcus gauvreauii* group, were positively correlated with nearly all HADG-associated metabolites; *Candidatus Saccharimonas* and *Succinivibrionaceae UCG-001* were positively correlated with more than 50% HADG-associated metabolites, and *Streptococcus* was positively correlated with ~ 35% HADG-associated metabolites, and those bacteria were negatively correlated with ≥ 50% LADG-associated metabolites (*p* < 0.05). In contrast, some LADG-enriched bacteria were significantly negatively correlated with ADG-associated metabolites (*p* < 0.05). For example, *Prevotella* was negatively correlated with isoleucyl-hydroxyproline, and valyl-hydroxyproline; *Prevotellaceae UCG-003* was negatively correlated with melibiose and maltotriose (*p* < 0.05).

### Rumen fluid microbiota features and milk yield in adult goats with varied growth rates during early life

**Fig. 7:**
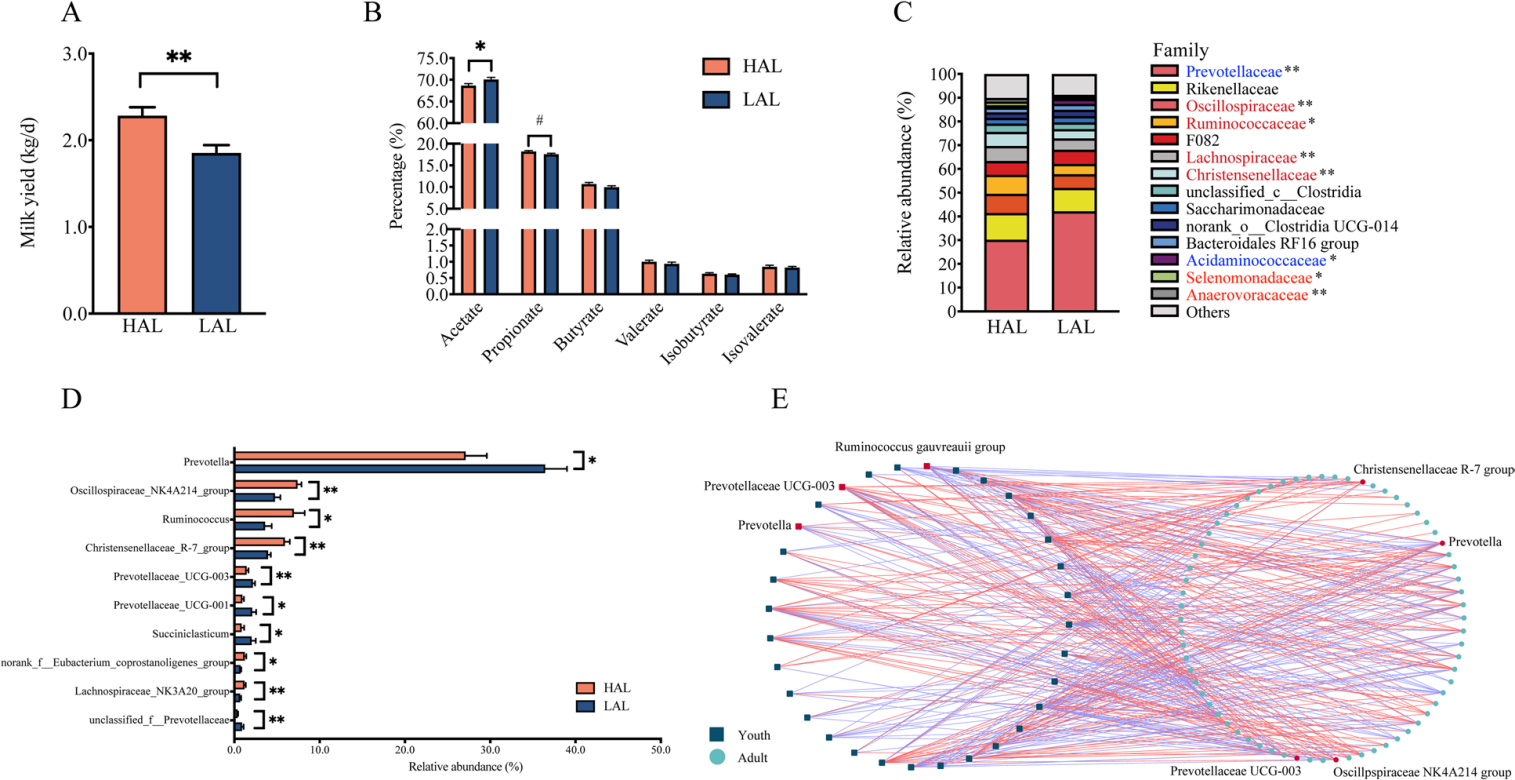
The influence of the rumen fluid microbiome of young goats on the rumen fluid microbiota composition and milk performance of adult goats.** A** Comparison of milk yield between the HAL and LAL groups. **B** The difference in the percentage of ruminal VFAs between HAL and LAL goats. **C** Distribution of abundant bacterial families.** D** Top 10 different genera identified by the Wilcoxon rank-sum test between HAL and LAL goats. Bars represent the mean ± SE. **E** Correlation networks showed association in the microbiota between young and lactating goats. Edge color represents either positive or negative associations between bacteria (*p* < 0.05). The genera of young goats with more than 5 edges (links) were retained. **A**,** B** Student’s *t* test, data are presented as the mean ± SE. **D** Wilcoxon rank-sum test. **E** Spearman’s rank correlation. ^#^ 0.1 < *p* < 0.05 *p < 0.05, **p < 0.01 HAL: lactating goats of HADG, LAL: lactating goats of LADG

To determine whether the rumen fluid microbiota of young animals has long-term impacts on rumen microbiota features and host performance, we then measured the milk production, rumen fermentation, and microbiota of HADG lactating goats (HAL, *n* = 13) and LADG lactating goats (LAL, *n* = 12). The growth rate of these young goats (*n* = 25) had a significant positive correlation with their subsequent milk yield (*r* = 0.36, *p* = 0.048). Moreover, compared to the LAL group, the milk yield, milk fat, protein, and lactose yields were significantly higher in the HAL group (*p* < 0.05; Fig. 7a; Fig. S4a). Compared to the LAL goats, the HAL goats also had a lower molar percentage of acetate, and acetate to propionate ratio (*p* < 0.05; Fig. 7b; Fig. S4b).

**Fig. 8:**
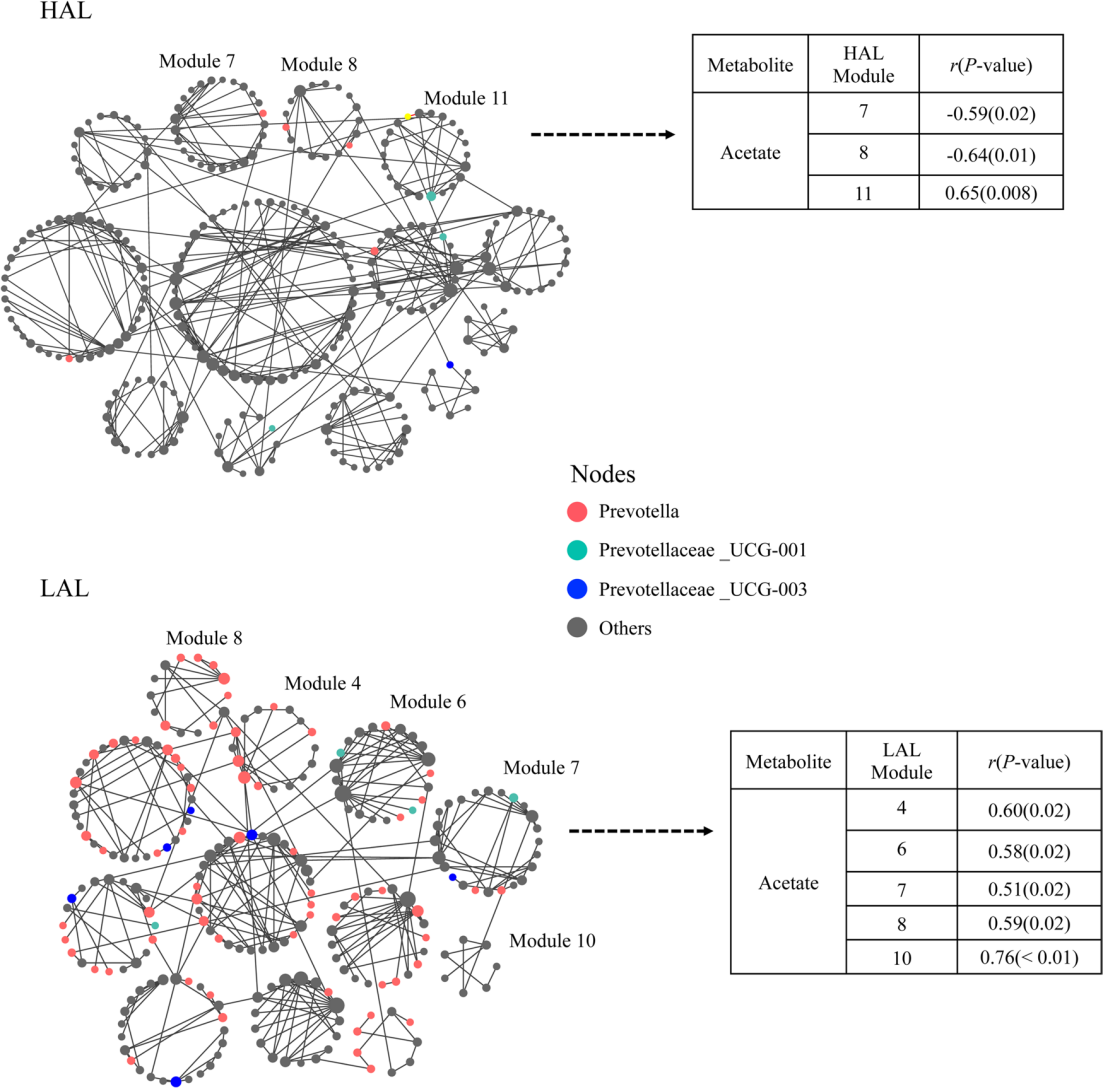
Co-occurrence network of rumen ASVs in HAL and LAL goats, and the network modules associated with the percent of acetate. Nodes represent an ASV, and only significant (Pearson’s correlation, *p* < 0.05) relationships are shown in solid lines HAL: lactating goats of HADG, LAL: lactating goats of LADG

Although there was no significant difference in Chao1 and Shannon indices of rumen fluid microbiota between these two groups (Fig. S5a, b), the microbiota profiles of the HAL group clustered significantly different from that of the LAL group (ANOSIM *R* = 0.178, *p* = 0.002; Fig. S5c). Moreover, the family *Prevotellaceae,* genus *Prevotella*, and *Prevotellaceae UCG-003* were significantly higher in the LAL group (*p* < 0.05; Fig. 7c, d). The abundance of *Oscillpspiraceae NK4A214* group, *Ruminococcus*, and *Christensenellaceae R-7* group were significantly higher in the HAL group (*p* < 0.05; Fig. 7c, d), with *Prevotella*, *Prevotellaceae UCG-003*, *Oscillpspiraceae NK4A214* group, and *Christensenellaceae R-7* group were the keystone bacteria in young goats (Table S4). Network module-trait relationship analysis revealed that in the LAL group, 5 modules (4, 6, 7, 8, and 10) were positively correlated with the molar percentage of acetate (*r* >|0.58|, *p* < 0.05; Fig. 8). The majority of nodes (ASVs) in two modules (4, and 8) belonged to the genus *Prevotella* (module 4: 6 out of 16, module 8: 7 out of 14). In contrast, in the HAL network, module 7 and module 8 were negatively correlated with the molar percentage of acetate (*r* <  − 0.59, *p* < 0.02).

### The rumen keystone bacteria of young goats further affected host rumen microbiota features

Furthermore, the correlation between the bacteria in the rumen of young goats and adult goats was identified (genera with relative abundance ≥ 0.1%; Fig. 7e; Table S8). Notably, some ADG-related bacteria in the youth period were significantly correlated with bacteria in adult goats, such as *Prevotella*, LADG-enriched bacteria in the youth period, had a positive correlation with the abundance of *Prevotella*, had a negative correlation with *Ruminococcus*, and *Christensenellaceae R-7* group (*r* >|0.4|, *p* < 0.05) in the lactation period. *Prevotellaceae UCG-003*, the LADG-enriched bacteria of young goats, was significantly correlated with more than 20 genera of adult goats, including *Oscillpspiraceae NK4A214* group, *Christensenellaceae R-7* group, and *Lachnospiraceae NK3A20* group (*r* >|0.5|, *p* < 0.05). *Ruminococcus gauvreauii* group, the HADG-enriched bacteria in the youth period, had a positive correlation with *Ruminococcus, Oscillpspiraceae NK4A214* group, *Christensenellaceae R-7* group in the lactation period, had a negative correlation with *Prevotella*, and *Prevotellaceae UCG-001* in the lactation period (*r* >|0.4|, *p* < 0.05).

### Rumen microbiota and metabolites of young goats with high prediction accuracy for ADG and milk production traits

**Fig. 9:**
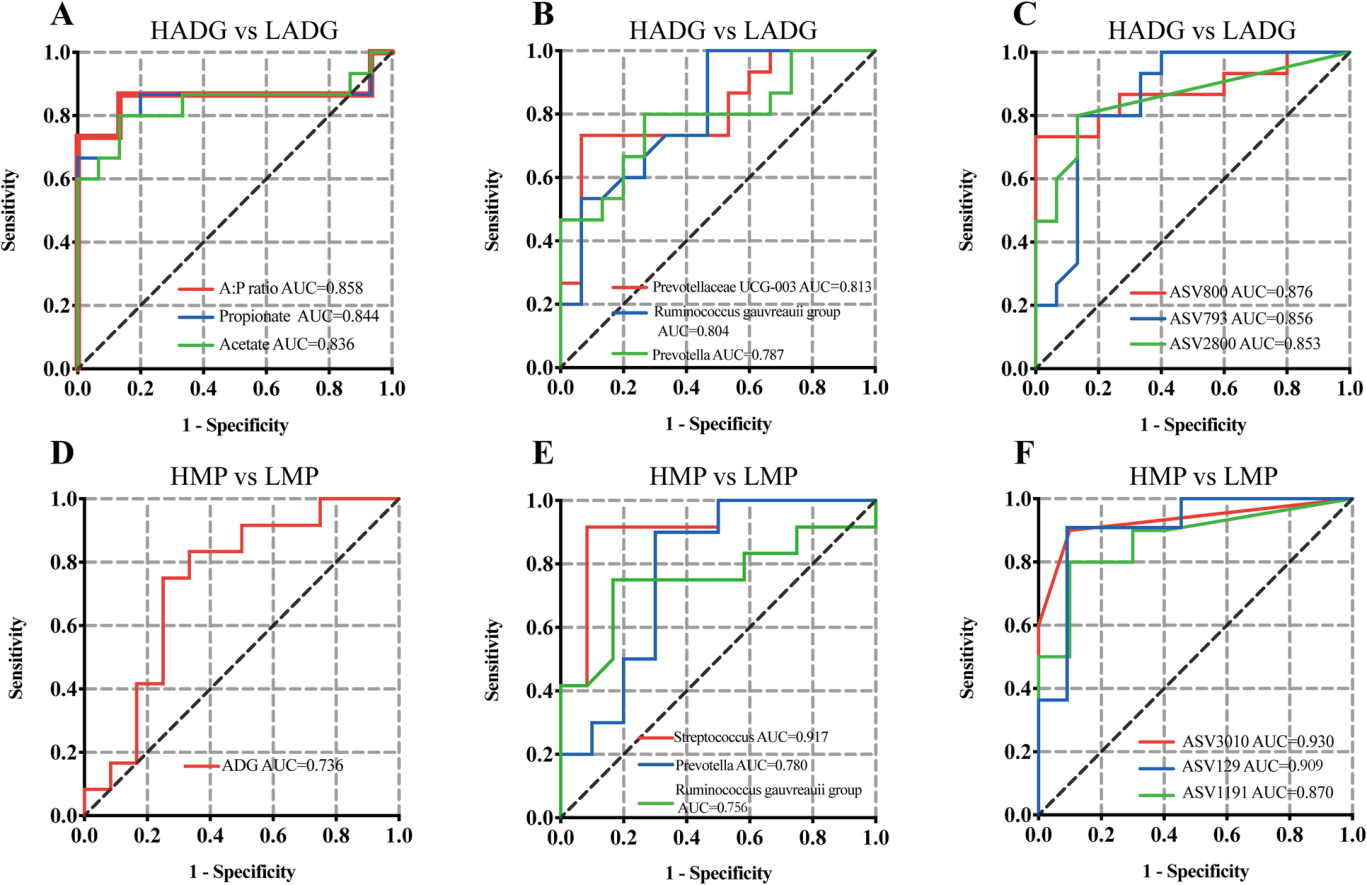
Prediction analyses based on the random forest model. Classification of host ADG using rumen metabolites (**A**) and microbiota (**B**,** C**) of young goats (*n* = 30), HADG vs LADG. Prediction of milk yield using ADG (**D**), and rumen microbiota (**E**, **F**) of young goats (*n* = 30), HMP vs LMP. ASV800: *Candidatus Saccharimonas*, ASV793: norank *F082*, ASV2800: *Prevotellaceae* UCG-003, ASV3010: *unclassified Oscillospirales*, ASV129: *Streptococcus*, ASV1191: *Prevotella *HADG: young goats with high average daily gain, LADG: young goats with low average daily gain, HMP: lactating goats with high milk yield, LMP: lactating goats with low-milk yield

We then explored whether differential rumen fluid microbiota and metabolites associated with ADG in young goats can be used to predict ADG in youth, and/or milk production in adult animals using the random forest model. Rumen VFAs, such as the acetate to propionate ratio, the molar percentage of acetate, and propionate, classified the HADG and LADG goats with high accuracy (AUC > 0.830; Fig. 9a). A total of 30 differential metabolites could classify high and low ADG goats with AUC > 0.80 (Table S7). Of these, isoleucy-tyrosine, L-lysopine, and 3-nonenoylglycine were the top three features with AUC > 0.90 of rumen metabolome profiles (Fig. S6). Notably, we found that *Prevotellaceae UCG-003*, *Ruminococcus gauvreauii* group, and *Prevotella* had AUC values of 0.813, 0.804, and 0.787 in classified HADG and LADG goats (Fig. 9b), and the top 3 ASVs (ASV800: *Candidatus Saccharimonas*, ASV793: *norank F082*, ASV2800: *Prevotellaceae UCG-003*) with AUC values > 0.850 (Fig. 9c).

Next, we explored whether ADG and rumen fluid microbiota of young goats could potentially serve as markers to predict adult milk yield. The results showed that the ADG of young goats had an AUC of 0.736 for classifying lactating goats with high- or low-milk yield (Fig. 9d). Rumen fluid bacteria, such as the top genus (*Streptococcus*) and ASV feature (ASV3010: *unclassified Oscillospirales*), had AUC values of 0.917 and 0.930, respectively, to predict milk yield. Moreover, the ADG-related genera, *Ruminococcus gauvreauii* group, and *Prevotella*, also showed AUC value > 0.750 (Fig. 9e, f) for milk yield prediction.

## Discussion

In the present study, a large number of goats under the same dietary conditions and management were used to untangle the relationship between the rumen fluid microbiome and host growth rate, and revealed the microbiota and its metabolites of young ruminants affected their ADG and could have long-term implications and predict milk production during first lactation in dairy goats.

First, the current study identified varied complex interactions among rumen fluid microorganisms, and keystone taxa as well as their relationships with rumen fermentation and ADG in young goats. Compared with the studies conducted in dairy cows, very few studies have explored the rumen microbial composition and function in dairy goats. These few studies revealed that rumen microbial communities of goats play important roles in feed degradation and rumen fermentation [27] and undergo significant changes in response to shifts in age and diet [28, 29]. Many recent studies have investigated the potential impact of the rumen microbiome on cattle phenotypes (such as feed efficiency and milk production) [9, 13]. However, the microbial community structure, composition, and function differ between goats and cattle [27, 30]. Henderson et al. [30] reported that microbial communities of goats clustered separately from cattle in the rumen, with bacteria being the main drivers behind the observed differences. For example, unclassified *Veillonellaceae* were proportionally more abundant in the rumen of goats, while the abundance of *Fibrobacter* was enriched in the rumen of cattle. This suggests that the influence of the rumen microbiome on goat growth could differ from that reported for cattle. The current study is a large-scale systemic study to reveal the rumen microbiome and its relationship with goat growth, and milk production traits.

Most previous studies on the biodiversity of microbial communities have been focused on the number of species and the abundance of species, but not interactions among species. However, microbiota interactions could be more important to ecosystem functioning than bacterial richness and abundance in complex ecosystems [31, 32]. Co-occurrence network analysis of the rumen microbiome of dairy cows revealed differential microbial interaction patterns between animals with different feed efficiency [33]. Previous studies in the rumen microbiome showed that the keystone species play an exceptionally important role in determining the structure and function of ecosystems [12, 34]. In the present study, we found that more network modules in LADG and LAL goats than in HADG or HAL goats were positively correlated with the molar percentage of acetate. Based on network topology, we also found that HADG-enriched *Candidatus Saccharimonas* (module hub), LADG-enriched *Prevotella* (network hub) and *Prevotellaceae UCG-003* (module hub) serve as keystone taxa of microbial networks in the rumen of HADG or LADG goats. These ADG-related keystone taxa together with non-ADG-related keystone taxa (*Oscillospiraceae NK4A214* group, and *Christensenellaceae R-7* group [module hubs and connectors]) may play important roles in microbiota structure and rumen function. From ecological perspectives, module hubs and connectors were close to generalists and network hubs were supergeneralists [35]. In this study, identifications of generalists and supergeneralists furthered the understanding of microbial community structure and differential microbial interactions, which play an empirical rather than theoretical role among microbial interactions [36]. Similar to our findings, a recent study found that *Christensenellaceae R-7* group, and *Prevotella* were the keystone species in the network, and they may play important roles in the rumen of yaks at different growth stages [34]. The present study is among the first to document the importance of bacterial interactions and keystone species in shaping the rumen microbial communities and rumen functioning of goats.

In the lactating period, these keystone bacteria, including *Prevotella*, *Christensenellaceae R-7* group, *Oscillospiraceae NK4A214* group, and *Prevotellaceae UCG-003*, were also different between the HAL and LAL groups. Some specific bacteria, such as *Prevotella*, and *Prevotellaceae UCG-003* of young goats, were significantly correlated with bacteria of adult animals, such as *Prevotella, Oscillospiraceae NK4A214* group and *Christensenellaceae R-7* group. Christensenellaceae, Oscillospiraceae, and Prevotellaceae and their genera were demonstrated to be keystone species for complex plant polysaccharides and were positively correlated with VFA concentrations [37–40]. These keystone species have similar reaction substrates and might regulate microbial interactions through nutritional supplementation or competition. Similar to our findings, Braga et al. [41] reported that microbes can interact with each other for the purpose of co-evolution leading to adaptation and specialization of certain microbial taxa, which promote future alterations in the microbial community. Recent studies found that increased growth and lactation performance could be achieved by increasing the abundance of rumen *Oscillospiraceae NK4A214* group and *Christensenellaceae R-7* group [42–44]. These genera have been reported to be associated with the degradation of a diverse array of structural carbohydrates as well as the production of propionate and butyrate [45, 46], which may provide more energy and a healthy rumen environment for host. Furthermore, *Prevotella* was the predominant, early colonizer and occupied various ecological niches within the rumen [47–49], and early arriving species exerted strong priority effects, having long-lasting impacts on the development of animal microbiomes [25].

In the present study, ADG-related genera played important roles in rumen fermentation (Fig. 6b, c) and had effects on goat growth. The abundance of *Prevotellaceae* family and its genera, which utilize various substrates (such as cellulose, starch, and protein) to mainly produce acetate and succinate [39, 40], have shown a negative relationship with goat growth rate. Moreover, the abundance of *Prevotella* was found to be negatively correlated with milk performance [8, 50], and feed efficiency [51] in ruminants. Indeed, our finding that the abundance of these *Prevotella* genera, and modules mainly composed of *Prevotella* have positive relationship with rumen acetate percentage provided further support for this notion (Fig. 4d, e; Fig. 6c; Fig. 8). However, in contrast to these findings, some studies also identified various *Prevotella* species associated with higher feed efficiency [52], and milk production [13, 53]. These conflicting results could be related to the large diversity of *Prevotella* with different functions, and the different metabolites shaped by microbe‒microbe interactions (the interaction between *Prevotella* with other genera). These findings suggested that strong microbial interactions of genus *Prevotella* with other others may increase rumen acetate production in LADG and LAL goats.

*Candidatus* saccharimonas, *Ruminococcus gauvreauii* group, *Streptococcus*, and *Succinivibrionaceae UCG-001*, HADG-related genera, also play important roles in feed digestion. *Streptococcus* and *Candidatus Saccharimonas* have been shown to be involved in amino acid biosynthesis and the metabolism of energy substrates [37, 54–56]. Furthermore, as a growth-related taxon in pigs [57], *Streptococcus* may also play an important role in promoting goat growth performance. Members of *Succinivibrionaceae* family compete with methanogens for hydrogen to produce succinate (a precursor of propionate) rather than methane [58], and were found to be associated with low methane yield [59], high ADG [60], and low abundance of methanogens [58] in ruminates. Meanwhile, our metagenome data revealed that fewer methanogens, such as *Methanobrevibacter smithii*, *Methanobrevibacter curvatus*, and *Methanoregula formicica*, were detected in HADG goats. Together, these bacteria are therefore candidates for further investigation to elucidate their association with growth, which might provide insight into whether these microbes could be manipulated to improve feed efficiency and the performance of goats.

Together, rumen keystone bacteria and early colonizers may exert priority effects, occupy ecological niches, and form complex microbial interactions with others, which will have long effects on microbiota succession, rumen fermentation, and host growth phenotypes. Feed intake is one of the key factors that affects animal growth performance, feed efficiency, and rumen microbiome. Long-term experiments should be conducted to further determine the relationship between feed intake, rumen microbiome, and feed efficiency of ruminants. Inoculating keystone species associated with feed efficiency or animal performance as precision probiotics in early life may modify microbiota colonization or composition, and subsequently improve animal performance. The present study investigated the potential role of rumen fluid microbiome in goats at early stage in regulating rumen fermentation and production performance in adult dairy goats. However, in addition to the liquid-associated microbiome, rumen particle- and epithelium-associated microbiome also play a key role in regulating rumen function [61, 62]. Future research activities will be carried out to comprehensively understand the role of different kinds of microbiome in the rumen, and how different members of the rumen microbiota may influence each other, and effective microbial manipulation tools and techniques.

There are considerable benefits associated with understanding rumen function, as rumen dynamics are almost solely responsible for providing nutrients to the host animal [63]. The genes encoding CAZymes involved in deconstructing carbohydrates, such as starch (GH13_39 and CBM41) and xylan (CE2 and CE3), and KEGG functions in carbohydrate metabolism were enriched in the rumen of HADG goats, including “TCA cycle, pyruvate metabolism, butanoate metabolism, and propionate metabolism.” The genes encoding relevant enzymes, such as EC2.7.1.11, which were involved in the glycolysis pathway, EC4.1.3.30, EC1.1.157, and EC1.2.7.1, which were involved in the pyruvate, propionate, and butyrate metabolism pathways, were enriched in HADG goats. EC1.2.13, which was involved in translating acetyl-Coa to acetate, was less abundant in HADG goats. These results indicated that the microbiome of HADG goats has a stronger ability to degrade carbohydrates to produce pyruvate, and subsequently used for more propionate and butyrate production, rather than methane and acetate biosynthesis. Together, these findings clearly indicated that the specific microbiome functional potential related to carbohydrate degradation and VFA biosynthesis significantly changes in goats with different growth rates, which advances the understanding of the functional roles of the rumen microbiome in contributing to rumen fermentation and goat growth.

The present study was one of the first to explore the relationship between rumen metabolites, microbiota taxa, and growth performance of dairy goats. These findings of rumen metabolite data are in good agreement with the rumen metagenome that more disaccharides, such as maltoriose, melibiose, and 3-galactosyllactose, which are intermediates in carbohydrate metabolism, and propionate and butyrate production were enriched in HADG goats, methane yield and acetate biosynthesis were enriched in LADG goats (Fig. 2 and Fig. 6). These disaccharides can be hydrolyzed to produce glucose for growth and development of the body [64]. Furthermore, in rumen VFA fermentation, propionate fermentation is the most energy efficient, due to assimilating energy from hydrogen and being the main precursor of gluconeogenesis in animals, whereas acetate production is accompanied by hydrogen production [65, 66]. For host animals, the energy recovery efficiency is increased by 9% when the substrate (glucose) is fermented into propionate but reduced by 22% and 38% when fermented into butyrate and acetate, respectively [67]. Indeed, studies have revealed that efficient animals tend to have higher ruminal propionate and butyrate concentrations [9, 68]. Together, our data suggest that the microbiome of HADG goats has a higher feed efficiency with a higher ability and efficiency to degrade carbohydrates to produce more propionate and butyrate, less acetate and methane, to support the host’s energy requirements. Furthermore, amino acids and peptides are extremely important factors affecting rumen microbial growth and microbial protein synthesis [69], in which microbial protein contributes 60–90% of the protein absorbed at the duodenum [8, 70]. Compared to the LADG goats, 3 amino acid metabolism pathways, taurine and hypotaurine metabolism, lysine degradation, and D-arginine and D-ornithine metabolism, were enriched in the HADG goat microbiome. We also found that some amino acids and peptides were enriched in HADG goats, including BCAAs and some EAAs, which (such as His, Trp, and Arg) are the limiting AAs for growing goats and cows [71, 72]. Moreover, we identified the associations between rumen microbiota and rumen microbial metabolomes and found that ADG-associated microbial taxa were significantly correlated with carbohydrate and protein metabolism. Overall, our data revealed that some specific rumen taxa, such as *Prevotella*, and *Streptococcus*, may play important roles in producing small molecule metabolites and contributing to goat growth, which has rarely been reported in dairy goats previously.

Furthermore, the development of sequencing techniques and machine learning methods facilitates the application of the microbiota and microbial metabolites to predict host phenotypes, including for the prediction of disease risk [73, 74] and performance [33]. In the present study, we found that rumen metabolites and microbes could predict host growth performance with high accuracy (Fig. 9a–c). Moreover, the rumen microbiota of young goats can also predict further milk yield with high accuracy (Fig. 9d,e). Notably, the random forest machine learning algorithm revealed that *Streptococcus* in young goats can be a key microbial marker that can differentiate high- and low-milk yield goats, with an accuracy of 91.7%. To our knowledge, it is still challenging to predict ruminant performance using only early-life microbial markers in practice. Further studies are required to integrate various key data, such as host genetic data, microbiome features, and metabolites, and find the best combined features to improve the accuracy and robustness of prediction, which could help us apply our findings in practice in the future.

The rumen microbial community is influenced by multiple factors, including diet, environment, and host genotype. In our study, all the goats were fed the same diet and raised under the same feeding and management regimes, the interanimal variations in the rumen microbiome may be directly or indirectly affected by animal genetics. Several studies have revealed that some rumen microbiota are heritable and associated with host phenotypes [12, 75, 76]. For example, a recent study found that *Prevotella* was related to several loci on cattle chromosomes 2, 6, 9, 19, 23, and 27 [76]. Future studies focus on revealing the relationship between rumen microbiota and genetic markers, which will help us use marker-assisted selection and management to improve feed efficiency, and animal performance.

## Conclusion

In summary, ADG-related microbiota and microbial interactions affect rumen fermentation and feed efficiency, and subsequently affect the energy and nutrients that are supplied to the host. Our study strengthens the notion that rumen microbial variations can lead to variations in feed efficiency, which will affect host performance. Overall, rumen microbiome features (such as diversity, structure, composition, and function) were different among young goats with different growth rates. Some bacteria and archaeal species, such as members of *Prevotellaceae* family, *Streptococcus*, and *Candidatus Saccharimonans*, were identified for their significant associations with animal growth. These rumen microbial variations contribute to carbohydrate and protein metabolism functions, and ruminal propionate, butyrate, and ruminal amino acid production in high ADG goats, which could provide more energy and nutrients for goats. Additionally, high ADG goats have more milk yield in the lactation stage. Some keystone bacteria may have long effects on microbiota succession, feed fermentation, and host phenotypes. The rumen microbiota of young goats identified by random forest analysis could be used as effective biomarkers for predicting animal performance (growth rate and milk yield). Thus, our results provide a deeper understanding of the potential influence of the rumen microbiome on feed efficiency and animal performance, highlighting the long-term role of keystone bacteria in microbe‒microbe interactions and in shaping microbiota composition and rumen fermentation patterns, and may aid in developing strategies to improve feed efficiency and animal performance.

## Methods

### Animals, grouping, and sampling

All the goats, which were fed the same diet, kept under the same conditions, and housed together during the whole experimental period, were raised in a Saanen goat farm in Baoji, Shaanxi (34° 41′ N, 109° 09′ E). The detailed feeding programs are shown and described in Supplementary Table S8. Animals that had been administered antimicrobial agents (antibiotics, antifungals or antivirals) within 3 months before sampling or had a history of an infectious disease or other force majeure were excluded from the experiment.

A total of 99 healthy female Guanzhong goats (born in late January 2020) were used for the experiment (Fig. 1). The birth weight of all goat kids was recorded immediately after birth. Herein, at the age of 6 months old (187.9 ± 0.3 [mean ± SE] days of age), the body weight of all selected goats was weighed at least two times, the mean body weight was calculated for each goat, and rumen fluid and blood samples were collected before the morning feeding. Individual weight gain was calculated as the difference between 6-month body weight and birth weight, and individual ADG was calculated as weight gain divided by the number of days.

The feed intake of HADG and LADG goats (*n* = 15 each group) was measured 2 weeks before weighing (174.2 ± 0.4 days). In brief, feed offered to and refused by each goat was recorded continuously for 7 days. The feed samples were dried at 65°C for 48 h to obtain the dry matter content of the ration. Daily dry matter intake (DMI) per goat was calculated by multiplying daily as-fed intake by the dry matter content of the ration. There was no significant difference in DMI between the HADG and LADG (1.03 ± 0.03 vs 1.01 ± 0.02, *P* = 0.735, *t* test).

Then, these dairy goats were naturally oestrus and mated in late September (8 months old). A total of 15 goats, who were not pregnant, or had been administered antimicrobial agents within 3 months before sampling, or had a history of infectious disease, or some other force majeure were excluded from the sampling. Then, since kidding in late February 2021, 84 remaining dairy goats entered the lactation period. The lactation performance of all dairy goats was recorded. Briefly, all 84 goats were milked twice daily at 0630 and 1600 h. The milk yield was recorded every 7 days over the first whole lactation period, the milk sample (2/3 from the morning and 1/3 from the evening milking were pooled as a daily sample) was collected for the milk composition analysis, and the average milk yield, milk composition of each goat was used for the following analysis. Meanwhile, the rumen fluid sample was also collected at 2 to 3 h after the morning feeding of the 23rd week of lactation (19 months old).

Briefly, rumen fluid samples were collected via esophageal tubing. The first 50 mL of rumen fluid was discarded to avoid saliva contamination, and the next 50 mL rumen fluid was strained through four layers of sterile cheesecloth under a constant flux of CO_2_.

According to the calculated ADG from birth to 6 months old, the 15 highest ADG goats of these 99 goats were selected as the HADG group (ADG: 132.5 ± 1.5 g/day), and the 15 lowest ADG goats were selected as the LADG group (ADG: 88.2 ± 1.2 g/day). After delivery of their newborn offspring kids, 84 of 99 lactating goats remained. Among these 84 lactating goats, 13 lactating goats of 15 HADG goats remained as the HAL group; 12 lactating goats of 15 LADG goats remained as the LAL group. Furthermore, in order to explore whether the rumen fluid bacteria, VFAs and ADG of young goats could serve as useful biomarkers to predict the milk yield in adult animals, the 15 lactating goats of these 84 lactating goats with the highest milk yield were selected and named the HMP group (average daily milk yield: 2.82 ± 0.04 kg/day), and 15 lactating goats with the lowest milk yield were selected and named the LMP group (average daily milk yield: 1.51 ± 0.03 kg/day), according to the milk yield recorded every 7 days over the first whole lactation period.

### *Estimation of methane production using an *in vitro* experiment using rumen fluid of young goats*

To estimate the ruminal methane production of young goats (6 months old) with different growth rates, rumen fluid samples of the HADG and LADG groups (*n* = 15 each group) were collected on the same day and used for the in vitro rumen fermentation study immediately.

The in vitro experiment was conducted following the procedure of Mauricio et al. [77], and artificial saliva (buffer) was prepared according to the formula provided by Menke et al. [78]. Briefly, under anaerobic condition, approximately 1.0 g total mixed ration (TMR) of young goats (DM basis, shown in Table S9) was weighed into individual 135 mL fermentation bottles, and then 20 mL collected rumen fluid from each goat, and 40 mL buffer was added into the fermentation bottle. Fermentation bottles, which contained the differential rumen fluid from different young goats of the HADG and LADG groups (*n* = 15 each group), were sealed with butyl rubber stoppers and aluminum crimp caps and placed in an incubator at 39°C for 24 h in a simulative incubator under anaerobic condition (ANKOM DAISY II, Ankom Technology Macedon, New York, USA).

At 4, 8, 12, 18, and 24 h of incubation, the total gas of each bottle was separately collected. The total gas of the same incubation tubes collected at different times was mixed. The volume of the total gas produced was determined using a glass syringe. Then, approximately 15 mL of gas sample was taken from the collected total gas and injected into gas chromatography to determine the CH_4_ concentration [79, 80], and the total CH_4_ production was calculated as CH_4_ concentration × total gas production. Furthermore, after 24 h of incubation, fermentation was terminated by placing bottles on ice. After opening the bottles, 5 mL fermentation broths were collected and stored at − 80°C until for VFA analysis.

### VFAs, amino acids, milk composition measurement

The concentrations of VFAs (acetate, propionate, butyrate, valerate, isobutyrate, and isovalerate) were determined using gas chromatography (Agilent 7820A, Santa Clara, CA, USA) with a capillary column (AE-FFAP of 30 m × 0.25 mm × 0.33 μm; ATECH Technologies Co., Lanzhou, China) according to the method described by Li et al. [81]. In brief, the thawed rumen fluid samples were centrifuged for 10 min at 16,000 × *g* at 4°C. Two milliliters of the supernatant were mixed with 25% metaphosphoric acid (400 μL). After standing for 4 h at 4°C, the mixture was centrifuged for 10 min at 16,000 × *g* at 4°C. Two hundred microliters of crotonic acid (10 g/L) were added to an aliquot (200 μL) of the supernatants and then filtered through a 0.45-μm filter. The injector and detector temperatures were set at 200°C and 250°C, respectively. The column temperature was increased from 45 to 150°C at 20°C/min and held for 5 min.

Rumen free amino acid abundance analysis was performed through separation and quantification by liquid chromatography‒mass spectrometry (LC‒MS) (Exion LC AC, QTRAP 5500, AB SCIEX, Framingham, MA, USA) with a phase column (4.6 mm × 100 mm × 2.7 μm Infinity Lab Poroshell 120 EC-C18, Agilent, Santa Clara, CA, USA) [82]. The MS is equipped with electrospray (ESI) as an ion source and is an ion mode of ESI + . The main parameters were as follows: curtain gas, 40 psi; collision gas, medium; ion spray voltage, 5500 V; temperature, 650°C; ion source gas 1, 60 psi; ion source gas 2, 60 psi. The LC conditions were as follows: column temperature, 30°C; flow rate, 0.8 mL/min; automatic sampler temperature, 15°C; injection volume, 5 μL; mobile phase A, 0.1% acetic acid + water; mobile phase B, 0.1% acetic acid + acetonitrile.

Milk samples were analyzed for fat, protein, and lactose using a milk composition analyzer (MilkoScan FT1, FOSS, Denmark).

### DNA extraction and 16S rRNA gene sequencing

Total DNA was extracted from rumen fluid samples of 99 young goats and 25 lactating goats (HAL: *n* = 13, LAL: *n* = 12; Fig. 1) using the QIAamp DNA Stool Mini kit (QIAGEN, Germany) according to the manufacturer’s protocol. The DNA concentration was measured with a Nanodrop-2000 (Thermo Fisher Scientific, USA), and the quality was assessed by 1% agarose gel electrophoresis. Bacterial 16S rRNA gene fragments (V3-V4) were amplified from the extracted DNA using the forward primers 338F (5′-ACTCCTACGGGAGGCAGCAG-3′) and the reverse primer 806R (5′-GGACTACHVGGGTWTCTAAT-3′). All amplicons were sequenced using the paired-end (2 × 300 bp) method on a MiSeq platform (Illumina, USA) following standard protocols [83].

The raw sequences were merged with FLASH (v1.2.11) [84] and quality filtered with fastp (0.19.6) [85]. Sequences were imported into QIIME2 v2021.8 for demultiplexing and the construction of an amplicon sequence variant (ASV) table using DADA2 [86]. Bacterial 16S ASVs were assigned a taxonomy using the SILVA database (version 138) as the reference. The relative abundance of a taxon in the sample was the fraction of the taxon observed in the ASV table relative to the sum of all observed taxa corresponding to the sample in the ASV table. Alpha diversity indices, including the Sobs, ACE, Chao1 richness estimate, and Shannon diversity index, were calculated using QIIME2, and analyzed at the ASV level. Principal coordinate analysis (PCoA) was performed based on Bray‒Curtis distance, and statistical significance was determined using analysis of similarities (ANOSIM) with 999 permutations at the ASV level. Taxa with relative abundance ≥ 0.1% for downstream analysis of correlation and comparison.

### Metagenome sequencing

DNA extract of HADG and LADG (*n* = 8 each group) was fragmented to an average size of approximately 400 bp using Covaris M220 (Gene Company Limited, China) for paired-end library construction. Individual sequencing libraries were prepared using the TruSeqTM DNA Sample Prep Kit (Illumina, San Diego, CA, USA). Metagenome library sequencing was performed on an Illumina HiSeq4000 platform (150 bp paired-end sequencing; Illumina Inc., San Diego, CA, USA).

Adapter sequences were trimmed off from the paired-end reads using SeqPre (v1.1). Low-quality reads (quality scores < 20 or length < 50 or having N bases) were removed using Sickle (v1.33). Host reads were filtered by aligning reads against the Capra hircus genome with BWA (v0.7.9a) and removing reads with high-scoring alignments host. These high-quality reads were then assembled into contigs using Megahit (v1.1.2). The assembled contigs were subjected to prediction of open reading frames (ORFs) using MetaGeneMark (v2.10) [87]. Non-redundant contigs were identified using CD-HIT (v4.6.1) at 95% sequence identity and 90% coverage [88]. The quality-filtered sequence reads were mapped to the representative sequences with 95% identity using SOAPaligner (v2.2.1) [89], and the gene abundance in each sample was calculated as reads per kilobase per million mapped reads (RPKM).

Representative sequences of non-redundant gene catalog were aligned to the NCBI NR database using Diamond (v0.8.35) for taxonomic annotations using the Best-hit method [90]. KEGG annotation was conducted using Diamond against the Kyoto Encyclopedia of Genes and Genomes database (v94.2). Carbohydrate-active enzyme annotation was conducted using hmmscan against the CAZy database [91]. All these databases had an E-value cut-off of 1e^−5^ while annotating ORFs.

### Analysis of rumen metabolome

The rumen fluid samples of young goats were thawed at 4°C and extracted using methanol/acetonitrile (1:1, v/v) buffer at 1:2 of sample: buffer. The mixtures were vortexed for 30 s, and then extracted in an ultrasonic bath (Kunshan Ultrasonic Instrument Co. Ltd., China) at 5°C with an ultrasonic frequency of 40 kHz for 30 min and incubated at − 20°C for 30 min. After centrifugation at 13,000 × *g* for 15 min at 4°C to precipitate the protein, the supernatants were transferred and dried using a vacuum evaporator. The concentrated product was resuspended in 100 μL of water/acetonitrile (1:1, v/v), and then subjected to LC‒MS analysis using an UHPLC system (Q-Exactive, Thermo Fisher Scientific, USA). Quality control was performed via a pooled QC sample by mixing equal volumes (20 μL) of each sample. Chromatographic separations were performed on an ACQUITY UPLC HSS T3 column (100 mm × 2.1 mm, 1.8 μm) (Waters Co., USA). The column temperature was maintained at 40°C, and the injection volume was 2.0 μL. Eluent A was prepared by mixing water/acetonitrile (5:95, v/v), and eluent B was prepared by mixing acetonitrile/2-propanol/water (47.5:47.5:5, v/v/v). The mass spectrometric data in both positive and negative modes were collected using an electrospray ionization source.

The LC‒MS data were processed using Progenesis QI software to extract raw peaks, filter and calibrate baseline, align peaks, deconvolute, identify peaks, and integrate peak areas. Rumen metabolite that was present in < 50% of samples or with a relative standard deviation > 30% were removed [92]. The metabolites were annotated into “carbohydrates and carbohydrate conjugates,” and “amino acids, peptides, and analogs” (subclass) using the human metabolome databases [93] for downstream analysis.

### Construction of microbial co-occurrence networks based on random matrix theory

Microbial co-occurrence networks were constructed using a random matrix theory (RMT)-based pipeline with default parameters as described by Deng et al. [94] to identify microbial interactions. Briefly, the data matrix of standardized relative abundance, multiplied by 10^6^ to satisfy the requirements of the pipeline, was uploaded to construct the network with default settings, including (1) the ASVs detected in ≤ 50% of all samples were excluded due to a drastic effect of ASV sparsity on the precision and sensitivity of network inference [95]; (2) only filling with 0.01 in blanks with paired valid values. The fast-greedy modularity optimization procedure was used for module separation. After modules were determined, eigengene analysis was used to reveal higher-order organizations in the network structure [94, 96]. In eigengene analysis, each module is represented by its singular value decomposition of the abundance profile called the module eigengene [96]. The within-module degree (Zi) and among-module connectivity (Pi) were calculated and plotted to generate a scatter plot for each network. The relationship between module-based eigengenes and environmental traits was analyzed using Pearson correlation coefficients. The visualization of the network structure was performed using Cytoscape v3.8.0. In this study, we used the simplified classification as follows: (i) peripheral nodes (Zi ≤ 2.5, Pi ≤ 0.62), which had only a few links and almost always to the nodes within their modules, (ii) connectors (Zi ≤ 2.5, Pi > 0.62), and (iii) module hubs (Zi > 2.5, Pi ≤ 0.62). Species with either a high value of Zi or Pi were generalists. These included module hubs, i.e., highly connected species linked to many species within their own module, and connectors highly linked to several modules. (iv) Network hubs or supergeneralists (Zi > 2.5, Pi > 0.62), acted as both module hubs and connectors [35]. These connectors, module hubs, and network hubs of the microbial network may play keystone roles in the microbial communities, which were called keystone species in this study.

### Construction of random forest classifier

The random forest package in R was used for the random forest analysis [97], with the rumen fluid bacteria, rumen metabolites, and ADG of young goats (HADG and LADG, HMP and LMP) being used as the inputs of the random forest model to classify high or low ADG goats, and/or predict milk yield in adult goats. Each genus, ASV from 16S rDNA sequence data (relative abundance ≥ 0.1%), metabolite from metabolome data, and each VFA were considered as a feature. The machine learning design was performed according to measure the description by Verhaar et al. [98]. To present overfitting, we used a nested cross-validation design in performing these models. In each of the 30 iterations, the dataset was randomly split into a test set containing 30% of the subjects and a training set with the remaining 70%. Within the training set, fivefold cross-validation was performed to optimize the model hyperparameters. The resulting model was evaluated on the test set, which yielded an area under the receiver-operator curve (AUC). These were recorded for each iteration and were averaged across 30 iterations. The random forest analysis was implemented in R using the randomForest package with default parameters. ROC curve results were plotted manually by the true positive rate against the false positive rate. ROC curves were constructed, and the AUC was used to designate the ROC effect.

### Statistical analysis

The 16S rDNA sequence data (rumen microbial alpha diversity, phyla, families, and genera) and metabolome data (KEGG pathways, KEGG enzymes, CAZymes, and rumen archaea) were compared between the two compare groups (HADG vs LADG, HAL vs LAL) which were compared using the Wilcoxon rank-sum test with the FDR adjusted *p* value < 0.05 considered significantly different. Phenotypic data (milking traits, and rumen VFAs) were compared between the two groups using *t* test, and *p* value < 0.05 was considered significant. Each metabolite from rumen metabolome data was compared using the Wilcoxon rank-sum test between two groups, with the FDR adjusted *p* value < 0.05, and the variable importance in projection (VIP) from orthogonal partial least squares discriminant analysis > 1 being considered significantly different metabolites.

Correlation analysis between rumen bacterial taxa, alpha diversity indices, rumen VFAs, AAs, and growth performance traits (*n* = 99) was performed using Spearman’s rank correlation, with coefficient >|0.2|, *p* value < 0.05 considered significant. Correlation analysis between ADG-related bacteria and metabolome profiles, ADG-related ASV and KEGG pathways (*n* = 16) was performed using Spearman’s rank correlation, with coefficient >|0.4|, as well as *p* value < 0.05 that was considered significant. Pairwise correlations (Spearman’s correlation, *p* < 0.05) were used to generate genus-level co-occurrence networks.

### Supplementary Information

**Additional file 1**.

**Additional file 2.**

## Data Availability

Raw sequencing data of all 16S rRNA sequences and metagenomes have been deposited into the NCBI Sequence Read Archive (SRA) under accession numbers PRJNA836638 and PRJNA836913, respectively.

## Abbreviations

ADG: Average daily gain
ANOSIM: Analysis of similarities
ASV: Amplicon sequence variant
AUC: Area under the receiver operating characteristic curve
BW: Body weight
CAZyme: Carbohydrate-active enzymes
CV: Coefficient of variation
DMI: Daily dry matter intake
FDR: False discovery rate
GH: Glycoside hydrolases
KEGG: Kyoto Encyclopedia of Genes and Genomes
PCoA: Principal coordinate analysis
PERMANOVA: Permutational multivariate analysis of variance
SE: Standard error
VFAs: Volatile fatty acids
WG: Weight gain
