# Supplementary material for: Multi-omics revealed the long-term effect of ruminal keystone bacteria and the microbial metabolome on lactation performance in adult dairy goats
Source: Microbiome. 2023 Sep 29;11:215. doi: 10.1186/s40168-023-01652-5 (PMC10540338; doi:10.1186/s40168-023-01652-5)
Supplement: Supplementary file 2 — Additional file 1. [file 40168_2023_1652_MOESM1_ESM.docx]

Figure S1. The difference in rumen fluid microbiota features between HADG and LADG goats. **A** The alpha diversity of ruminal microbiota between HADG and LADG goats (Wilcoxon rank-sum test). **B** PCoA plot based on Bray-Curtis distance comparing ruminal microbiota composition. Significantly different family (**C**) and genera (**D**) between HADG and LADG goats based on Wilcoxon rank-sum test (relative abundance ≥ 0.1%), % the relative abundance of the taxa. The bars represent mean ± SE. **p* < 0.05, ***p* < 0.01.

HADG: young goats with high average daily gain, LADG: young goats with low average daily gain.

Figure S2. The top 5 significantly different rumen fluid archaea between HADG and LADG goats based on metagenome analysis (Wilcoxon rank-sum test, *p* < 0.05).

HADG: young goats with high average daily gain, LADG: young goats with low average daily gain.


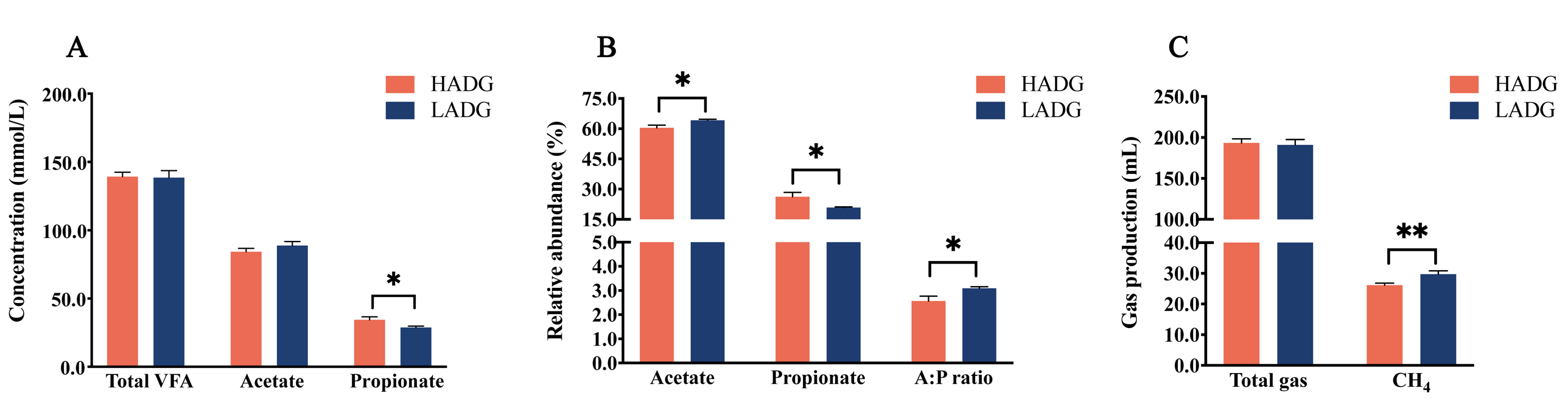


Figure S3. *In vitro* measurements show phenotype differences in VFAs (**A**,**B**) and methane production (**C**) between HADG and LADG goats. The bars represent mean ± SE, Student’s t test: **p* < 0.05, ***p* < 0.01.

HADG: young goats with high average daily gain, LADG: young goats with low average daily gain.

Figure S4. The difference in milk production (**A**), and acetate to propionate ratio (**B**) between HAL and LAL goats. Data are presented as the mean ± SE. Significant differences were test by Student’s t-test, **p* < 0.05.

HAL: lactating goats of HADG, LAL: lactating goats of LADG.





Figure S5. The difference in rumen fluid microbiota diversity and composition between HAL and LAL goats. **A** Chao1 index, and **B** Shannon index, Data are presented as the mean ± SE. **C** PCoA clustering analysis of rumen bacterial communities via Bray-Curtis distances, Distribution of abundance bacterial phyla (**D**). Significant differences were test by Wilcoxon rank-sum test. **p* < 0.05, ***p* < 0.01.

HAL: lactating goats of HADG, LAL: lactating goats of LADG.


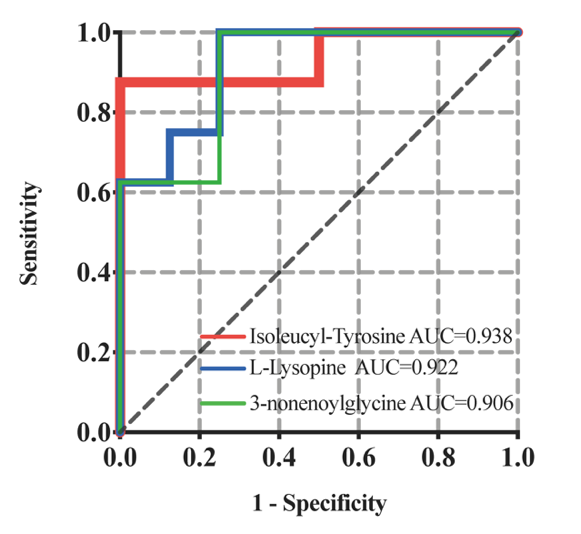


Figure S6. Classification of host ADG using the rumen metabolome profiles based on random forest model.
